# Editorial Notes

**Published:** 1917-04

**Authors:** 


### Editorial Notes.

**Death of three Past Presidents.**

It is with a deep sense of the loss to the medical profession in Bristol and the neighbourhood that we have to record within the short space of time covered by one issue of our *Journal* the loss of four of those who have enriched the work of the Bristol Medico-Chirurgical Society, three of them having held the office of President. We do not propose to touch on personal characteristics or qualifications here, for these our readers naturally turn to our Obituaries; but while each one has left his mark not only on our local medical life and in our affections, the names of Mr. Lansdown and Dr. Munro Smith seem to stir up distant

**Memories of the last century.**

Mr. R. G. Poole Lansdown followed his father, who practised in Bristol, and in the early sixties lived in Portland Square, on the side facing the church. He used to drive a yellow barouche, followed by a Dalmatian dog, and many of us can remember in the last quarter of the past century the late Dr. Henry Marshall's Dalmatian, without which his carriage seemed incomplete. But in the early sixties Portland Square was in the height of fashion, and many of the wealthier merchants and most successful practitioners lived there, besides which it was the place where the polling booths were located during Parliamentary elections, in those days not at all tame affairs.

Facing Mr. Lansdown's house, and next but one to the church, was another surgeon, Mr. William Ormerod, the father of our late colleague Mr. Henry Ormerod, of Westbury-on-Trym, who in turn has been succeeded by his son Mr. H. L. Ormerod, now in our midst. Another old Bristol doctor also lived in the Square, Dr. Fendick, whose son later practised in Clifton; and at the time we refer to Mr. W. D. Wills, the original member of the well-known firm, lived in Portland Square. His brother's (Mr. H. O. Wills) house was in Somerset Street. Again our President's (Professor Edgeworth) name comes before us, for though his father practised before him in Cotham, his grandfather dwelt hard by Portland Square, at the bottom of Paul Street.

The loss of Lieut.-Colonel Munro Smith similarly recalls to us the name of his father, Mr. William Smith, Surgeon, who in 1848 practised in Park Street, and was a friend of Joseph Williams, who was in practice near Portland Square. Joseph Williams, an uncle of the Editor of this *Journal*, was a friend of Mr. William Smith. He devoted himself to the medical care of the poor stricken in the cholera epidemic, and was at supper with Mr. Smith the evening before he himself was attacked, and soon after succumbed to cholera. Joseph Williams was accorded a public funeral, and a beautiful memorial was placed over his grave in Arno's Vale Cemetery by the subscriptions mostly from the poor citizens, who recognised that he had given his life for them and theirs. An uncle of Mr. William Smith's wife, Mr. Isaac Leonard, Surgeon, lived in Brunswick Square in 1847, and was the father of Mr. Crosby Leonard, one of Bristol's noted surgeons.

These old-time memories bring up the name of the second Dr. Edward Long Fox, who descended from the Quaker family of Falmouth. True, his virile mentality would have abhorred the latter-day breed now mistermed "conscientious" objectors, but in this connection we may fittingly recall a very interesting episode towards the close of the eighteenth century. The great-grandfather of Dr. Long Fox, a certain Joseph Fox, of Falmouth, had shares in ships that turned privateers in the war with France. As a conscientious objector to a war of that character, he declined his share in the profits, amounting to a large sum, and on the conclusion of peace he sent his son, Edward Long Fox (who became Physician to the Bristol Royal Infirmary in 1786), to Paris to restore to the French owners his share of what he deemed to be ill-gotten gains. The French Emperor was struck by his remarkable consistency, and conferred an honour, which, however, he declined on principle.

The CONSCIENTIOUS OBJECTOR, how different and degenerate is he to-day! He stands aloof when, without provocation, his brother is ruthlessly shot down, his wife and daughters murdered, or worse still, carried off to a slavery of the utmost depths of degradation; and not only leaves his fellows to give life and limb in defending the defenceless, but considers it beneath his Christian conscience, forsooth, to give a cup of water or bind up the wounds of those who are stricken while defending the objector's hearth and home, his wife and daughters, and precious self. We have yet to meet the objector who is consistent enough "to sell all his goods and give to the poor," or even to refuse all war profits on the goods he sells or the wage he earns. To medical men who, without invoking God and the Bible, as do the Kaiser and the conscientious objector, never heedlessly turn aside from healing the sores of even the most degraded sufferers, though their pains may be the direct result of their own misconduct, the cold and cruel disdain of the phylacteried Pharisee for the noblest of our race is altogether incomprehensible; yet maybe the feeding of swine, or similar work of national importance, suits better men of their kidney: or should we say "liver"?

This is your creed, to "turn the other cheek,"
Then turn it to your Master's stinging cord,
"Begin to be astonished," all ye meek,
Who do not cleanse His temple for your Lord.

Christ's word forbids you to defend the weak?
Stay wrong on land and horror on the sea?
Nay, let Himself your bitter judgment speak,
"Ye helped not them? Depart, ye helped not Me."

*G. I. W.*

**Honours.**

We offer most cordial congratulations to the following colleagues and confrères, in every case conferred since our last issue, for work of exceptional merit:—

Colonel James Swain, on whom His Majesty has conferred the Companionship of the Order of the Bath, Military Division. He is at present visiting military hospitals and attending a special conference at the front in France.

Lieutenant-Colonel Michell Clarke has been elected a member of the Council of the Royal College of Physicians, London, a distinction not often falling to the lot of provincial Fellows. The University of Bristol must be gratified to find their Pro-Vice-Chancellor's meritorious work and personal qualifications have been recognised in this appointment.

Captain R. A. Fuller, R.A.M.C., of Long Ashton, has been decorated with the Military Cross, awarded "for conspicuous gallantry and devotion to duty. He led stretcher parties and tended the wounded under intense fire, displaying great courage and determination throughout the operations."

Captain V. B. Green-Armytage, R.A.M.C., has done excellent work in connection with the expeditionary forces in Mesopotamia, and has been decorated by H.M. the King of Serbia with "the Order of the White Eagle, with Swords" for his medical services at Kut.

**Ministry of Health.**

We trust that the belief that Lord Rhondda will propose the creation of a Ministry of Health is well founded, for the proposal will certainly be welcomed by the medical profession if the powers conferred are practicable and in good hands. A Ministry of Health is not altogether a new proposal, for it has been urged before, though never with such authority as Lord Rhondda can bring to bear. And although, we understand, it will be brought forward as a war measure, and for the duration of the war, we have no doubt whatever that the Ministry of Health will become a permanent office of our Government. The appreciation of the work of the general practitioners, and the necessity for their work and interests being represented on equal terms with other aspects and departments of medical work, shows statesmanship and a grasp of the essential problems which such a Ministry ought to grapple with, so that we may look forward with confidence to its recognition as a factor of prime importance in national development.

*　　*　　*　　*　　*

**Plague Outbreak "Nipped in the bud."**

A concise and pithy article by the Medical Officer of Health for Bristol, Lieut.-Colonel D. S. Davies, appears in this issue. Those who have any gift of imagination will realise how immense a service has been rendered by skilful applications of scientific knowledge and the untiring energy of Colonel Davies, and of all those who worked in conjunction with him, otherwise the outbreak would have very rapidly got beyond control, and spread far and wide into many districts where no such intricate machinery exists for preventing a further extension. Bristol, like Liverpool, London, and other great ports, has thus to render services national quite as much as, if not more than, local. But this is by no means the first occasion that our M.O.H. has done brilliant work. Yet such a service is—like virtue—its own reward.

Let us see how the financial burden is distributed. We understand that the Bristol Health Committee were involved in an expenditure of no less than £4,000 on account of the preventive measures that had to be taken, and yet only £500 is to be contributed by the Local Government Board. The Ministry of Health will do useful work if more equitable arrangements are instituted, for it is difficult to understand why Bristol ratepayers should spend such large amounts in preventing a national outbreak, which would involve untold misery and immense cost to the nation. How curious it seems to be informed that the Board contended that it was the duty of Bristol to pay the whole cost, and that this grant must not be taken as a precedent. We hope not indeed !

It would be just as reasonable for the Admiralty to look to all seaport towns to provide all the cost of coast defence, and then contribute a small portion of the cost of an actual enemy attack.

**Sir Alfred Keogh, G.C.B.**

We offer our hearty congratulations to the Director-General of the Army Medical Services on the very high honour of the Grand Cross of the Bath recently conferred upon him. His most successful development of the Army Medical Services during war time is a magnificent contribution to the fighting power of the Empire, and very rare are the men who combine with such a charming yet withal tremendously forceful personality the necessary intellectual qualifications, the three essential qualifications of the born organiser.

**Medical Students and the War.**

SOME remarkable and interesting statistics furnished by the General Medical Council to the *British Medical Journal* afford food for reflection. These show that the actual number of male students in attendance on courses of instruction in preparation for medical degrees or diplomas are as follows:—

| | *First year.* | *Second year.* | *Third year.* | *Total.* |
|---|---|---|---|---|
| England (including London) and Scotland .. .. .. | 968 | 609 | 355 | 1,932 |
| Ireland .. .. .. .. | 481 | 378 | 217 | 1,076 |

In other words, the Irish school male students are more than half the number of those in all the schools of England and Scotland combined.

Yet when we turn to the *Medical Directory* we find that the numerical summary of the medical profession for 1917 shows that there are 30,838 in London, Provinces, Wales, and Scotland, and 3,193 in Ireland; that is to say, of qualified practitioners (including women) the proportion was less than one-ninth from Irish schools as compared with British, while the male students are now in the proportion of more than one-half in Irish schools as compared with British!

**Restrictions on the use of Medicinal Glycerine.**

THE Ministry of Munitions request us to announce that owing to additional demands for Glycerine for war purposes it has become necessary to place further restrictions on the issue of Medicinal Glycerine, and that supplies in future will be reserved for the manufacture of the preparations of the British Pharmacopœia, and for such uses of special importance as may be sanctioned by the Ministry of Munitions. These supplies will, however, be small, and must be used with the utmost economy.

Applications for permit to obtain supplies should be addressed to: The Director of Propellant Supplies, 32 Old Queen Street, Westminster, S.W., and should give the following particulars:—

1. Quantity applied for.
2. Stock of Glycerine held.
3. Purpose for which supply is required. (In case of extra British Pharmacopœia preparations, formulæ should be given.)
4. Applicant's average yearly consumption of Glycerine for above purposes.
5. Name and address of proposed suppliers.

The Medical Profession have been informed of the need for economy in prescribing Glycerine, and it is anticipated that the requirements for dispensing will be greatly reduced. The stocks of Glycerine in the hands of Pharmacists should be sufficient to meet these reduced requirements, and therefore no Glycerine will be issued for dispensing meantime.

The surplus stocks held by Pharmacists, and all stocks held by retailers who are not in a position to use them for these restricted purposes, should be disposed of either to other Pharmacists who are short of stock, or to wholesale houses for making B.P. preparations.

